# The Effect of Low-Magnitude Low-Frequency Vibrations (LMLF) on Osteogenic Differentiation Potential of Human Adipose Derived Mesenchymal Stem Cells

**DOI:** 10.1007/s12195-017-0501-z

**Published:** 2017-08-07

**Authors:** Monika Marędziak, Daniel Lewandowski, Krzysztof A. Tomaszewski, Krzysztof Kubiak, Krzsztof Marycz

**Affiliations:** 1 Faculty of Veterinary Medicine, University of Environmental and Life Sciences, Norwida 31 St, 50-375 Wrocław, Poland; 2 Institute of Material Science and Applied Mechanics, University of Technology, Smoluchowskiego 25 St, 50-370 Wroclaw, Poland; 3 Department of Anatomy, Jagiellonian University Medical College, Kopernika 12 St, 31-034 Kraków, Poland; 4 Department of Experimental Biology, University of Environmental and Life Sciences, ul. Norwida 27B, 50-375 Wrocław, Poland; 5 Wrocławskie Centrum Badan EIT+, Stablowicka 147 St, 54-066 Wroclaw, Poland

**Keywords:** Stimulation, Low-magnitude low-frequency vibrations, Vibration generator, Adipose derived mesenchymal stem cells, Osteogenesis, Bone loss

## Abstract

**Introduction:**

In the current study, we investigated the effect of low magnitude, low frequency (LMLF) mechanical vibrations on the osteogenic differentiation potential of human adipose derived mesenchymal stem cells (hASC), taken from elderly patients.

**Methods:**

During 21 days in osteogenic culture medium, cells were periodically exposed to three different frequencies (25, 35 and 45 Hz) of continuous sinusoidal oscillation, using a vibration generator. We measured cell proliferation, cell morphology, calcium and phosphorus deposition using Almar Blue assay, fluorescence microscopy, scanning electron microscopy, and a EDX detector, respectively. Osteogenic differentiation was measured by assessing protein and mRNA levels. Osteogenesis was confirmed by detection of specific markers with alkaline phosphatase and enzyme-linked immunosorbent assays for: bone morphogenetic protein 2 (BMP-2), osteocalcin (OCL) and osteopontin (OPN).

**Results:**

We found that 25 Hz vibrations had the greatest impact on hASC morphology, ultrastructure, and proliferation. We observed the formation of osteocyte- and hydroxyapatite-like structures, an increased quantity of calcium and phosphorus deposits, and increased differentiation in the stimulated groups.

**Conclusions:**

Our findings suggest that LMLF vibrations could be used to enhance cell-based therapies for treatment of bone deficits, particularly in elderly patients, where the need is greatest.

## Introduction

Bone tissue engineering is a rapidly developing field of regenerative medicine. This rapid expansion is in part driven by the ever-growing increase in the number of patients who stand to benefit from these treatments. These include patients who have sustained bone injuries and tissue loss due to trauma, cancer, osteonecrosis, osteoarthritis, and the ever growing population of elderly patients in whom the bone’s natural regenerative capacities are reduced, resulting in altered healing properties.^30^

While many treatments are available, the gold standard in reconstructive orthopedics continues to be vascularized or non-vascularized autologous bone grafts. This treatment consists of harvesting bone grafts from other parts of the patient’s own skeleton and transplanting it directly into the non-healing bone defect.^26^

Tissue engineering approaches, in many ways, simulate the bone grafting technique, in that they deliver different combinations of bone and vessel forming cells, cytokines, and 3D scaffolds directly into a defect, thus helping restore the bone’s natural regenerating ability. Many different cell-cytokine-scaffold combinations have been tried, both in preclinical, and, more recently, clinical trials.^1^ Finding the ideal bone substitute is the ultimate goal of tissue engineering, however, this continues to be a challenge. Attempts include the use of a variety of scaffolds such as 3D printed materials^33^ or hydrogels^16^ in combination with gelatin,^13^ hydroxyapatite, bone morphogenic protein-2 (BMP-2),^25^ calcium, phosphate, and hyaluronic acid.^27^ However, with all of these combinations, adding mesenchymal stem cells (MSC) and/or endothelial progenitor cells (EPC) to the mix^38^ seems to be essential. MSCs have been shown to have regenerative and anti-inflammatory properties, are able to differentiate into several different cell lineages, and, perhaps most importantly, are able to self-renew.^8^

For the treatment of bone defects, MSCs are typically harvested from the bone marrow. However, more recently, MSCs have been successfully obtained from adipose tissue. Adipose derived mesenchymal stem cells (hASC) have some important advantages over their bone marrow derived counterparts. Namely, they are abundant, are easily harvested, and cause less donor site morbidity than bone marrow-derived MSCs.^2^,^39^ Despite these advantages, recent reports suggest that advanced donor age is associated with a decrease in hASC proliferation and differentiation potential.^15^,^20^,^43^ This represents a major limitation to use of hASCs, as orthopedic injuries increase with advancing age and are most prevalent elderly populations.

To address this shortcoming, investigators have attempted different methods aimed at enhancing proliferation and differentiation of stem cells harvested from elderly donors. These methods include stimulating stem cells by exposing them to static magnetic fields,^19^ electric currents,^5^ and even both pulsed^22^,^42^ and continuous^12^ vibration stimulation.

These different forms of vibration stimulation have been shown to enhance and or modify cell proliferation and differentiation into different cell lineages, such as adipocytes,^40^ chondrocytes^24^ and osteocytes.^34^ This same phenomenon has been described in naturally occurring vibrations generated by working muscles, which impacts bone mineral mass, size, and gross structural properties.^9^,^28^ In addition to these naturally occurring, high-magnitude low-frequency vibrations (HMLF), vibrations of different magnitudes and frequencies have also been studied. Both high-magnitude high-frequency (HMHF)^31^,^40^ and low-magnitude low-frequency (LMLF) vibrations^12^,^35^,^36^ have been found to also impact bone material properties. In 2010, Ozcivici *et al*.^32^ in *in vivo* experiments, demonstrated that LMLF vibrations enhanced proliferation activity and osteogenic differentiation in mouse bone marrow-derived stromal cells. Enhancement of osteogenic differentiation potential of MSCs may strongly depend on up-regulation of particular integrins, that are activated by various biomechanical signals like for example heterodimeric adhesion proteins, consisting of linked α and β subunits. These adhesion receptors are mediated in cell interactions with extracellular matrix (ECM) and adjacent cells during morphogenesis. During the commitment of MSCs to the osteoblastic lineage a crucial role is played by upregulation of single subunits—αV, β3, α5, and the formation of integrin receptors α5β1 and αVβ3.^6^ However, the other integrins are still poorly investigated, especially in the context of their expression in differentiated precursor cells additionally stimulated by various types of external mechanical or others signals. Besides, the integrin receptors mediated osteogenic differentiation of MSCs, mechanotransduction have been showed to be an important factor that promotes osteogenesis. Nikukar and his colleges, have showed, that in particular nanoscale sinusoidal mechanotransducive stimuli called by them “nanockiging” (10–14 nm displacements at 1 kHz) promote osteoblastogenesis in human mesenchymal stem cell cultures.^29^

## Materials and Methods

To assess the effects of vibration stimulation on hASCs osteogenic differentiation potential *in vitro*, cells were cultured for 21 days in osteogenic conditions. During this time, the cells were exposed to 25, 35, 45 Hz (experimental groups) vibrations or no vibrations (control groups) for 10 min, every day (from day 0 to 21). The morphology, viability, and osteogenic differentiation potential of hASCs under the influence of the above mentioned frequencies was investigated.

### Ethics

All cell handling procedures described herein were performed in accordance with the ethical standards laid down in the 1964 Declaration of Helsinki and its later amendments, and were approved by the Local Bioethics Committee of Wroclaw Medical School (registry number KB-177/2014). All cell donors gave written informed consent prior to inclusion into the study.

### Cell Isolation Protocol

Subcutaneous adipose tissue was collected from six elderly patients (mean age ± SD 69 ± 1 year) during total hip arthroplasty. The tissue samples were placed in sterile Hank’s Balanced Salt Solution (HBSS) (Sigma Aldrich, Germany) and the hASCs were isolated under aseptic conditions, according to a previously described protocol.^7^,^18^,^44^ Isolated cells from each donor, were then pooled into one stock and after first passage the MSCs were used for vibration application. In brief, after washing with HBSS supplemented with 1% antibiotic-antimitotic solution (Penicillin/Streptomycin/Amphotericin B, Sigma Aldrich, Germany), the tissue was cut into pieces with surgical scissors, digested with 1 mg/mL collagenase type I (Sigma Aldrich, Germany), and incubated for 30 min at 37 °C. Tissue homogenate was centrifuged at 1200×*g* for 10 min and the supernatant was removed. The pellet containing cells was resuspended in culture medium and placed in a cell culture flask.

### hASC Characterization by FACS

Immunostaining and flow cytometry analyzes (FACS) were performed to detect and confirm the presence of specific cell surface antigens characteristic for hASCs. All mouse antibodies used [CD 29-PE (BD 562801), CD 34-PE-Cy7 (BD 560710), CD 44-APC (BD 559942), CD 45-PerCP (BD 557235), CD 73b-FITC (BD), CD 90-APC-Cy7 (BD 561401), CD 105- Percp-Cy5.5 (BD 560819) and streptavidin (BD 554066)] were purchased from BD Biosciences (USA). Fluorochrome-conjugated mouse immunoglobulin was used as isotype control. Single cell suspensions of hASC were subsequently analyzed on a Becton–Dickinson FACSCalibur flow cytometer to obtain at least ten thousand cells. Samples were analyzed by FlowJo software (TreeStar, USA).

### hASC Characterization by Multipotency Assay

To determine the multipotent character of isolated cells, hASCs were divided into two groups for culturing for 14 days. The first group was cultured in adipogenic conditioned media (StemPro^®^ Adipogenesis Differentiation Kit, Life Technologies, Poland), while the second group was cultured in chondrogenic conditioned medium (StemPro^®^ Chondrogenesis Differentiation Kit, Life Technologies, Poland). In both groups, the cells were seeded in concentration of 8 × 10^3^ cells per well. The media were changed every second day. After the culture period, the cells were fixed with 4% paraformaldehyde and stained with Oil Red O (3% solution in isopropanol) and Safranin O (0, 1% solution in distilled water) to show adipogenic and chondrogenic character, respectively, of the differentiated cells.

### Cell Culture for Vibration Application

Cells were maintained at constant conditions in an incubator (37 °C, 5% CO_2_ and 95% humidity) throughout the experiment. The primary culture was plated in a T-75 culture flask and cultured in Dulbecco’s Modified Eagle’s Medium (DMEM, Sigma Aldrich, Germany) with F-12 Ham nutrient, 10% Fetal Bovine Serum (FBS, Sigma Aldrich, Germany) and 1% PSA solution. The medium was changed every second day. Before being exposed to vibrations, cells were passaged three times using Trypsin–EDTA solution, according to the manufacturer’s instructions (Life Technologies, Poland), after reaching approximately 90% confluence.

Cells were divided into four groups based on exposure to different levels of vibration: (1) no vibrations (controls), (2) 25 Hz vibrations, (3) 35 Hz vibrations, and (4) 45 Hz vibrations. Each group was seeded in separate culture plates for ease of placement on the vibration generator. Cells were seeded on 24-well culture plates at a concentration of 8 × 10^3^ cells per well. Cells were cultured for 1 day in standard medium, after which the medium was replaced with media to osteogenic conditions (StemPro^®^, Osteogenic Differentiation Kit, Life Technologies, Poland) and changed every second day.

Each group was treated with the same magnitude (0.3 g) of LFLM vibrations for 10 min every day during the 21-days culture period. The vibrations treatments were applied outside the cell incubator. The control group was also placed outside the cell incubator each day for 10 min, however with no vibration application.

### Vibration Generator

**Figure 1:**
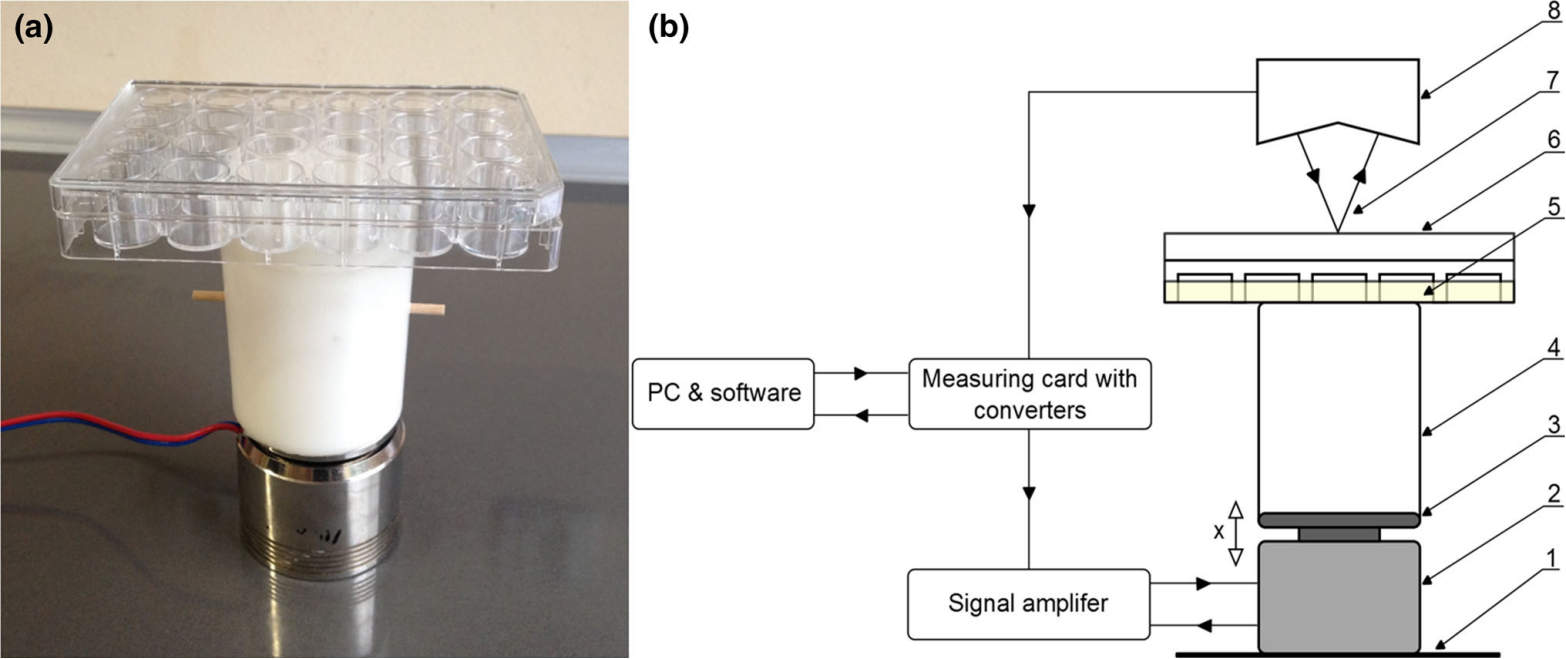
Vibration generator (a) with a schematic (b) denoting its individual parts and movement: x—direction of movement, 1—base, 2—electro-magnetic actuator with coil, 3—culture plate holding platform, 4—polyethylene spacer, 5—cell culture medium, 6—24-well cell culture dish, 7—laser beam, 8—laser displacement sensor head.

The vibrations in the experiment were generated by a specially designed electromagnetic device that generates harmonic sine waves of a given frequency and amplitude. The design of the device is similar to an acoustic loudspeaker, with the exception that the part that moves is the holding plate, which is linearly displaced in relation to a stationary base (Fig. 1).

The frequency and magnitude of the produced vibrations can be adjusted in the same way the sound level may be adjusted with a loud speaker. Displacement of the culture dish is measured with a laser displacement sensor (Keyence LK-G157) and the acceleration signal is calculated using the following formula:

$$ x = A\sin \left( {\omega t} \right) $$

$$ \ddot{x} = - A\omega^{2} \sin \left( {\omega t} \right) $$

where: x = displacement, $\ddot{x}$ = acceleration, *A* = amplitude of displacement, $\omega$ = frequency of vibrations ($\omega = 2\pi f$), *f* = frequency, *t* = time, and $A\omega^{2} =$ amplitude of acceleration.

A 10 cm polyethylene spacer located between the actuator and the culture plate, serves to isolate the cell culture plate from possible alternating magnetic fields generated by the electro-magnetic actuator.

### Proliferation Potential of hASCs

The cell proliferation factor (PF) was measured at day 2, 4, 8, 10, 12, 14 and 18 using Almar Blue, TOX-8 assays (Sigma Aldrich, Germany) according to manufacturer’s instructions. This was performed by replacing the medium with a 10% resazurin-based dye (Almar Blue). After 2 h incubation at 37 °C, the absorbance of the supernatant was measured (SPECTRO StarNano, BMG Labtech, Germany) at 600 nm wavelength, with a distraction of 690 nm background absorbance. The PF is an arbitrary unit that assigns a proliferation rate of the experimental groups compared to the control group, whereby a PF equal to 1, represents the same rate as the control.^23^ An online calculator (http://www.doubling-time.com/compute.php) was used to estimate cell amount and population doubling time (PDT).

### Evaluation of hASC Morphology

To evaluate the nuclei and f-actin distribution after 7 days of vibrations application, diamidino-2-phenylindole (DAPI, 1:1000) (Sigma Aldrich, Germany) and atto-488-labeled phalloidin (1:800) (Sigma Aldrich, Germany) staining was performed.^14^ Briefly, cells were fixed in 10% paraformaldehyde for 45 min and permeabilized with 0.2% Tween for 15 min. After fixation, cells were stained with atto-488-labeled phalloidin for 40 min or DAPI for 5 min. All fluorescence staining was performed in the dark and observed and digitalized using an inverted fluorescence microscope (AxioObserverA1, Zeiss, Germany) and a Cannon Power Shot digital camera, respectively.

To observe osteogenic nodules after 21 days of osteogenic culture, Alizarin Red staining was used. Alizarin Red dye (2% solution in distilled water) was applied for 10 min to visualize extracellular calcium deposits. Observations were performed using light microscopy (AxioObserver 1, Zeiss, Germany) and recorded using a digital camera (Cannon PowerShot).

Ultrastructure cell morphology was assessed using a scanning electron microscope (SEM) (EVO LS15, Zeiss, Germany). After fixation and dehydration in a rising ethanol series (concentrations 50–100%, every 10 min), cells were splattered with gold and observed using a SE1 detector, at 10 kV of filament’s tension. To measure hydroxyapatite-like structures on the surface of the osteoblast precursors, SEM (EVO/Analytics/MesureSys) was used. Measurements were performed on six different osteo-nodules in each culture plate. Additionally, the analysis of calcium and phosphorus depositions in investigated groups during the osteogenic differentiation was carried out using SEM–EDX technique as described earlier.^41^ A Quantax detector (Brüker) was used for the analysis, with the parameters of 10 kV of filament tension. From each sample six measurements were performed. The values obtained were presented as a weight percentage (wt%).

### Evaluation of Osteogenic Differentiation Markers on Protein and mRNA Level

To measure osteogenic differentiation, ALP activity and quantitative ELISA were used to detect human bone morphogenetic protein 2 (BMP-2), osteocalcin (OCL) and osteopontin (OPN). Supernatants from the 21-day culture medium were collected and analyzed for extracellular activity of alkaline phosphatase (ALP, Abcam—ab83369) and levels of BMP-2 (R&D Systems, DBP200), osteopontin (OPN, EIAab^®^—E0899h) and osteocalcin (OCN, EIAab^®^—E0471h). Samples were prepared in duplicate, using 100 µL of supernatant diluted two-fold. The fold changes are normalized according to standard curve. ALP activity (μmol/min/mL or U/mL) in the test samples is calculated as:

$$ {\text{ALP activity}} = B/(\Delta T \times V) \times D $$

where *B* = amount of pNP in sample well calculated from standard curve (*μ*mol), Δ*T* = reaction time (min), *V* = original sample volume added into the reaction well (mL), *D* = sample dilution factor.

Osteogenic differentiation was confirmed by measuring the expression of genes specific for osteogenesis on the mRNA level. After 21 day of culture, cells were homogenized with TRI Reagent (Sigma Aldrich, Germany) and a single-step RNA isolation method, as previously described by Chomczyński and Sacchi,^4^ was performed. RNA diluted in DEPC-treated water was analyzed for concentration and purity by means of nanospectrophotometry (VPS biowave II). Removal of genomic DNA (gDNA) was done using a DNase I RNase-free kit (Thermo Scientific, USA). A total of 1000 ng RNA was used for each reaction. Complementary DNA (cDNA) was synthesized with Moloney Murine Leukemia Virus Reverse Transcriptase (M-MLV RT, Life Technologies) and oligo(dT)15 primers (Novazym). The qRT-PCR mixture contained 50 ng cDNA, 500 nM forward and reverse primers, and SensiFast SYBR & Fluorescein Kit SYBR Green PCR Master Mix (Bioline). The primer sequences are presented in Table 1.

**Table 1:** Sequences of primers used for gene amplification.

Sequences of primers			
Gene	Abbreviation	Primer	Sequence 5′–3′
Glyceraldehyde-3-phosphate-dehydrogenase	GAPDH	Forward	GTCAGTGGTGGACCTGACCT
		Reverse	CACCACCCTGTTGCTGTAGC
Collagen type I	Col-I	Forward	GTGATGCTGGTCCTGTTGGT
		Reverse	CACCATCGTGAGCCTTCTCT
Bone morphogenic protein 2	BMP-2	Forward	ATGGATTCGTGGTGGAAGTG
		Reverse	GTGGAGTTCAGATGATCAGC
Alkaline phosphatase	ALP	Forward	CGCGCTTGTGCCTGGA
		Reverse	CCTGCTTTATCCCTGGAGCC
Osteocalcin	OCN	Forward	ATGAGAGCCCTCACACTCCTC
		Reverse	CGTAGAAGCGCCGATAGGC
Osteopontin	OPN	Forward	AAACGCCGACCAAGGTACAG
		Reverse	ATGCCTAGGAGGCAAAAGCAA
Integrin α3	ITGA3	Forward	ATCTTGAGAGCCACAGTCA
		Reverse	CTGGGTCCTTCTTTCTAGTTC
Integrin α4	ITGA4	Forward	AATGGATGAGACTTCAGCACT
		Reverse	CTCTTCTGTTTTCTTCTTGTAGG
Integrin α5	ITGA5	Forward	ACTAGGAAATCCATTCACAGTTC
		Reverse	GCATAGTTAGTGTTCTTTGTTGG
Integrin αV	ITGAV	Forward	GGAGCACATTTAGTTGAGGTAT
		Reverse	ACTGTTGCTAGGTGGTAAAACT
Integrin β3	ITGB3	Forward	CTGCTGTAGACATTTGCTATGA
		Reverse	GCCAAGAGGTAGAAGGTAAATA
Integrin β5	ITGB5	Forward	GAAGGGTTGCCCTCCAGA
		Reverse	GCTTGAGCTTCTCTGCTGTT

The reactions were conducted with CFX Connect™ Real-Time PCR Detection System (Bio-Rad, USA) under the following conditions: initial enzyme activation at 95 °C for 2 min, followed by 45 cycles of denaturation at 95 °C for 30 s, annealing for 30 s with the temperature dependent on the primer sequences (60 °C GAPDH; 61.5 °C Col-I; 67.1 °C BMP-2; 60 °C ALP; 64.8 °C OPN; 67.5 °C OCL, Integrin α3 52 °C; Integrin α4 58 °C; Integrin α5 52 °C, Integrin αV 56 °C, Integrin β3 52 °C; Integrin β5 60 °C) and extended at 72 °C for 30 s with a single fluorescence measurement. The expression level of each gene was normalized for the expression level of the housekeeping gene glyceraldehyde-3-phosphate dehydrogenase (GAPDH). The values obtained for each gene was divided by the value corresponding to GAPDH.

### Statistical Analysis

All collected data were analyzed using Microsoft Excel 2013 and GraphPad Prism 5. The one-way analysis of variance (ANOVA) with *post hoc* Dunnett’s test was used to determine statistical significance, with *p* values lower <0.05 considered statistically significant.

## Results

### hASC Characterization

**Figure 2:**
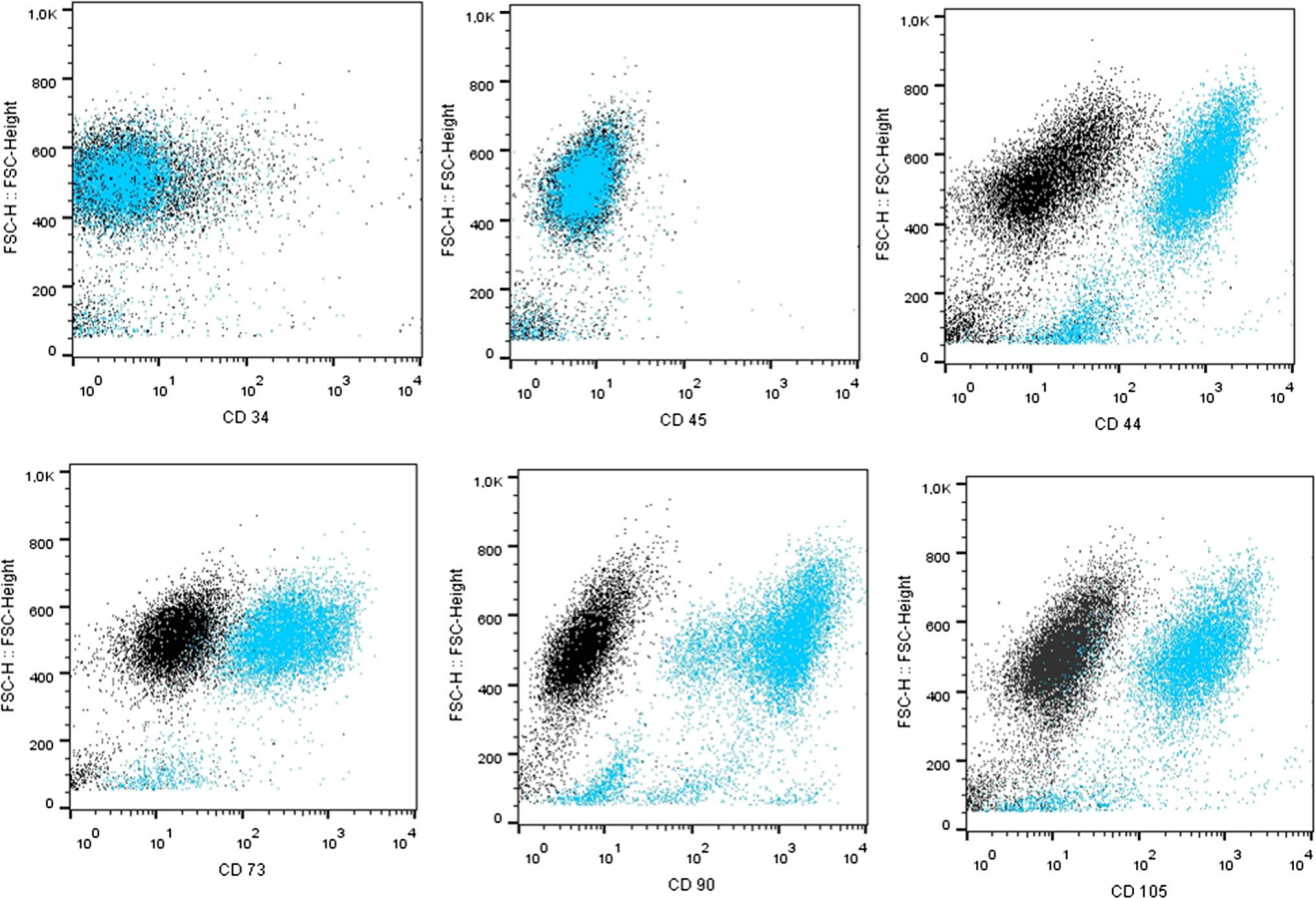
Representative data from flow cytometric immunophenotyping of hASC. The black dots represent isotype control IgG expression and blue dots depict marker expression. hASCs were positive for CD44, CD73b, CD90, CD105, and negative for the leukocyte common antigen CD45 and hematopoietic lineage marker CD34. *FSC* Forward Scatter.

Immunophenotypic characterization of hASCs obtained from the patient samples confirmed the presence of mesenchymal markers (CD29, CD44, CD73, CD90) and excluded hematopoietic origin (CD45, CD34) of the obtained cells (Fig. 2).

**Figure 3:**
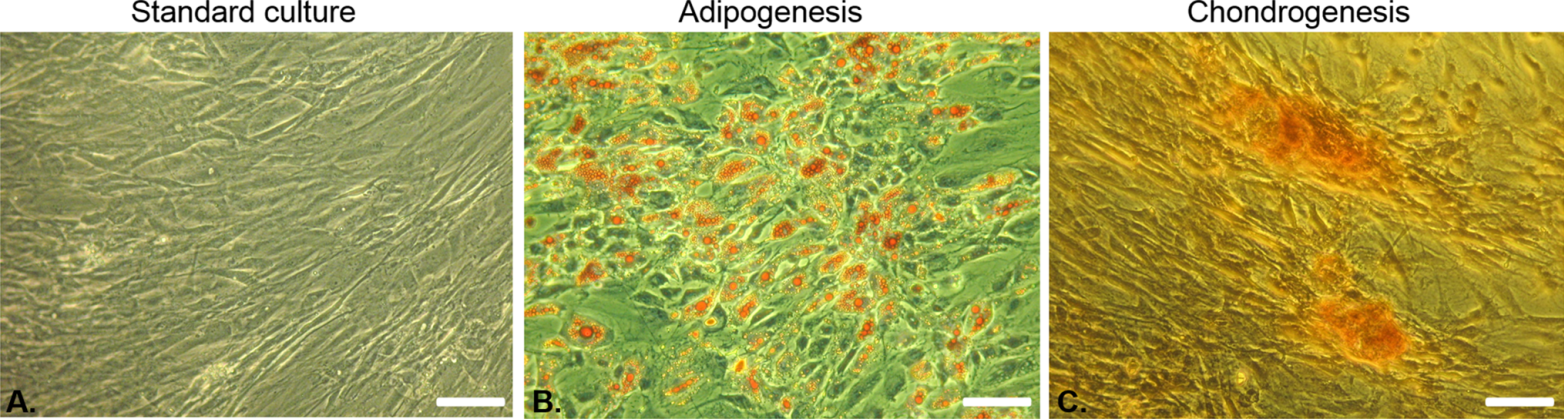
Multipotency of isolated ACS. (a) Cells cultured in normal medium. Oil Red O staining of adipocytes (b) and Safranin O staining of chondrogenic nodules (c) (magnification 100×, scale bar = 40 *µ*m).

Positive adipogenic (Fig. 3b) and chondrogenic differentiation (Fig. 3c) indicated the multipotent character of the cells in comparison to the standard culture (Fig. 3a). Lipid droplets of adipocytes were visualized with Oil Red O staining (Fig. 3b), while chondrogenic nodules were visible after Safranin-O staining (Fig. 3c).

### Cell Proliferation

**Figure 4:**
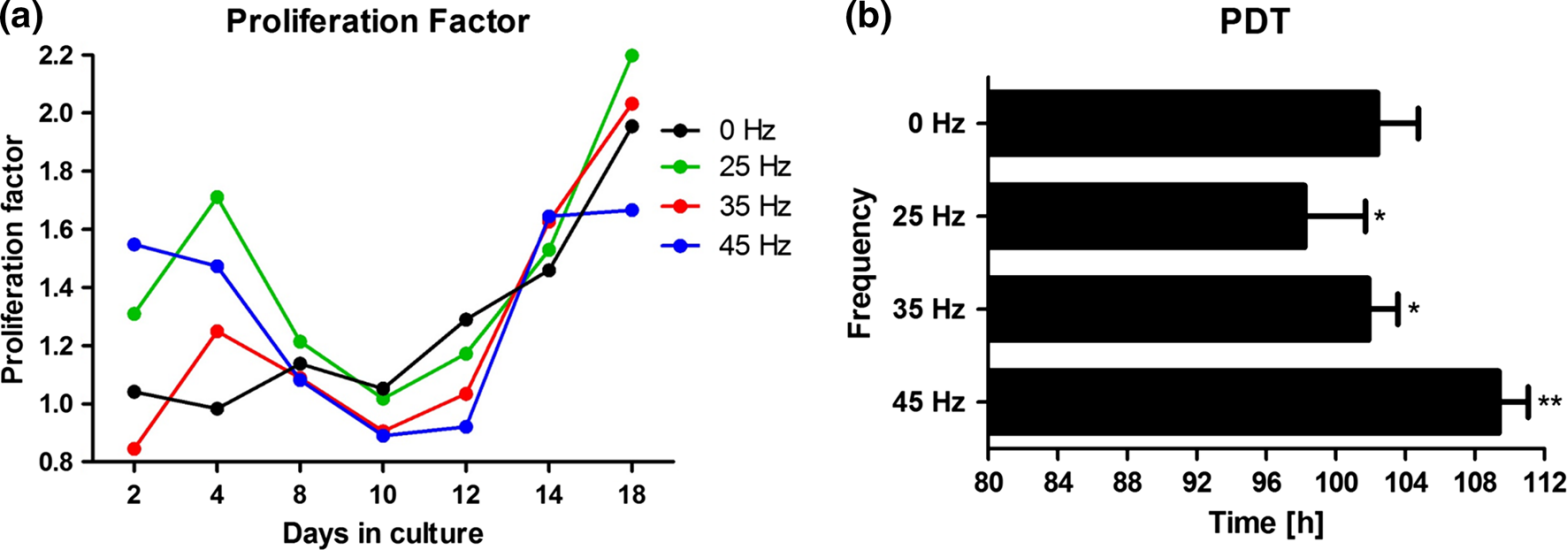
Proliferation factor (a), population doubling time (b) during osteogenic differentiation. *p value < 0.05, **p value < 0.01, all groups vs. control culture. Proliferation factor calculated as arbitrary unit based on number of cells in comparison to control. Proliferation factor equal to 1, represents the same rate as the control. Values above 1 indicate on increase cells viability, whereas below 1 as decrease.

In cells exposed to 25 Hz vibration, the PF at day 18 was higher, and their population doubling time (PDT) was significantly (*p* < 0.01) lower as compared to controls (Fig. 4). In cells exposed to 35 Hz vibration, the PF at day 18 was higher than controls but lower than that of the group receiving 25 Hz. Additionally, their population doubling time (PDT) was significantly (*p* < 0.05) lower than controls. This difference in PF between stimulated and non-stimulated cells declined after 18 days (Fig. 4a).

In cells exposed to 45 Hz vibration, the PF at day 18 was lower than in all other groups, including control (Fig. 4a). Moreover, their population doubling time (PDT) was longest in comparison to the other groups (Fig. 4b).

### hASC Morphology

**Figure 5:**
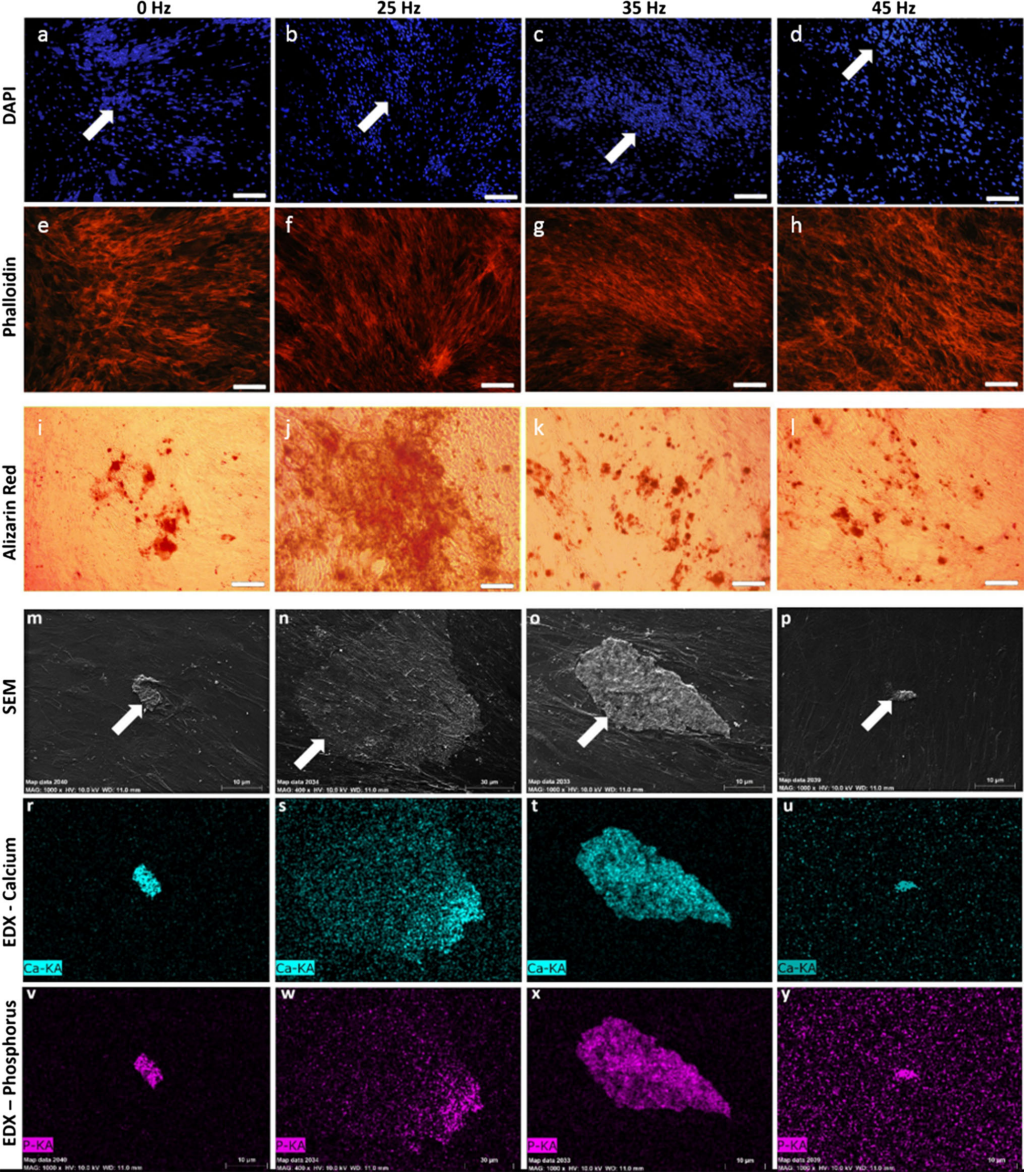
DAPI (a–d) and Phalloidin (e–h) staining of osteocytes, Alizarin Red staining of calcium deposition (i–l) (magnification 50×, scale bar = 600 *µ*m), SEM (m–p) and EDX images of hydroxyapatite-like structure formation (r–y). Significantly greater osteogenic nodule, and calcium and phosphorus deposits were observed in groups stimulated with 25 Hz vibration (magnification 1000×, scale bar = 10 *µ*m, for 25 Hz: magnification 400×, scale bar = 30 *µ*m). *KA* K alpha, name of the series of emission in the EDS spectra.

In the last day of osteogenic conditioned culture, the presence of osteogenic nodules, as well as hydroxyapatite-like structures, were observed in all tested the groups (white arrows), indicating a successful osteogenic process (Fig. 5). However, the most abundant osteogenic nodules, that were created by an extracellular matrix rich in hydroxyapatite-like structures, were observed in the 25 Hz vibration group (Figs. 5b, 5f, 5j, and 5n) as compared to the 35 and 45 Hz vibration groups, as well as the control group (Fig. 5). For 25 Hz group magnification of SEM photographs was higher to better show the osteo-nodules formatted in by cells stimulated with this frequency.

**Figure 6:**
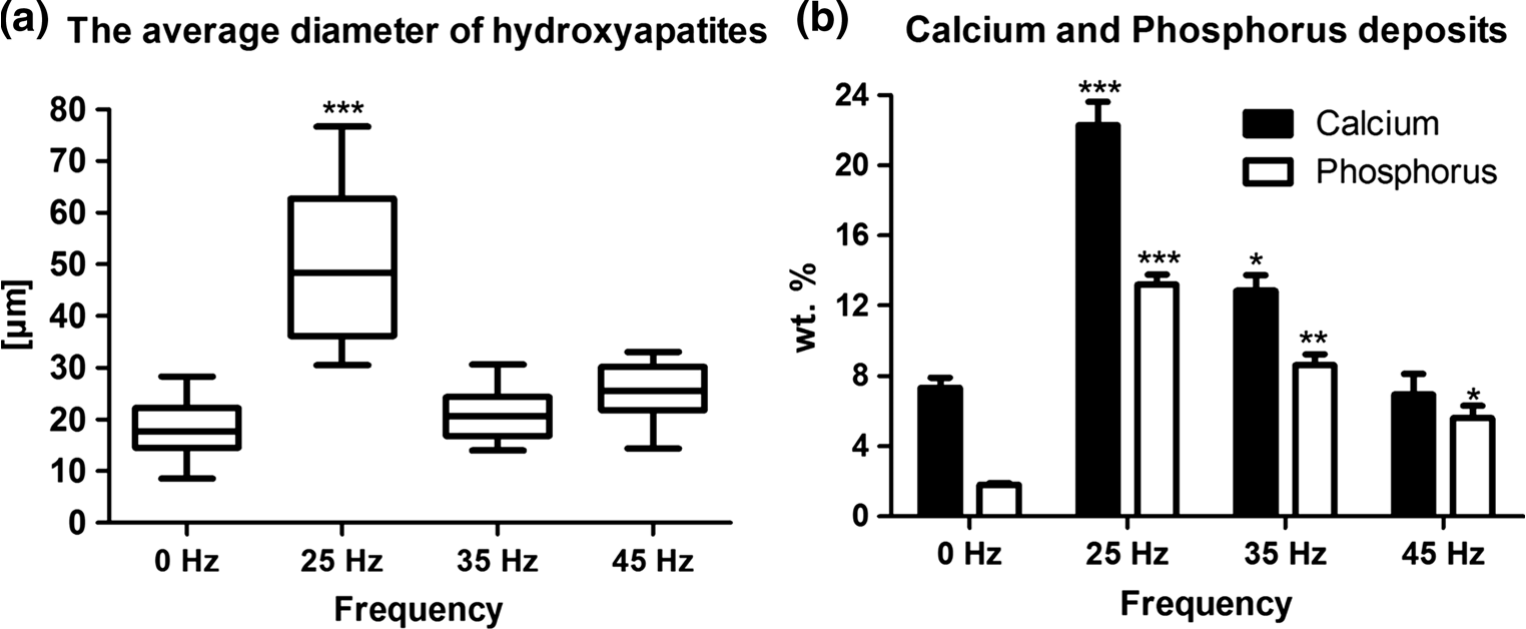
The average diameter of hydroxyapatite-like structures, measured by SEM analytical toll (a), calcium and phosphorus deposits, measured by EDX software (b). *p < 0.05, **p < 0.01, ***p < 0.001, all groups vs. control culture.

Cells cultured under 25 Hz vibrations formed nodules with a significantly higher diameter than samples cultured with other frequencies vibration groups and control group (Figs. 5r–5y and 6a). The quantitative measurement of calcium and phosphorus deposits in extracellular matrix (ECM) formed during osteogenic differentiation of hASCs indicated that matrix rich in calcium and phosphorus was produce in all investigated groups. In comparison to control, the amount of phosphorus in ECM of cultures subjected to 23, 35 and 45 Hz vibrations was significantly increased. The calcium content was also elevated, however only when cells were propagated on 25 and 35 Hz vibrations in comparison to control (Fig. 6b).

### Osteogenic Differentiation

**Figure 7:**
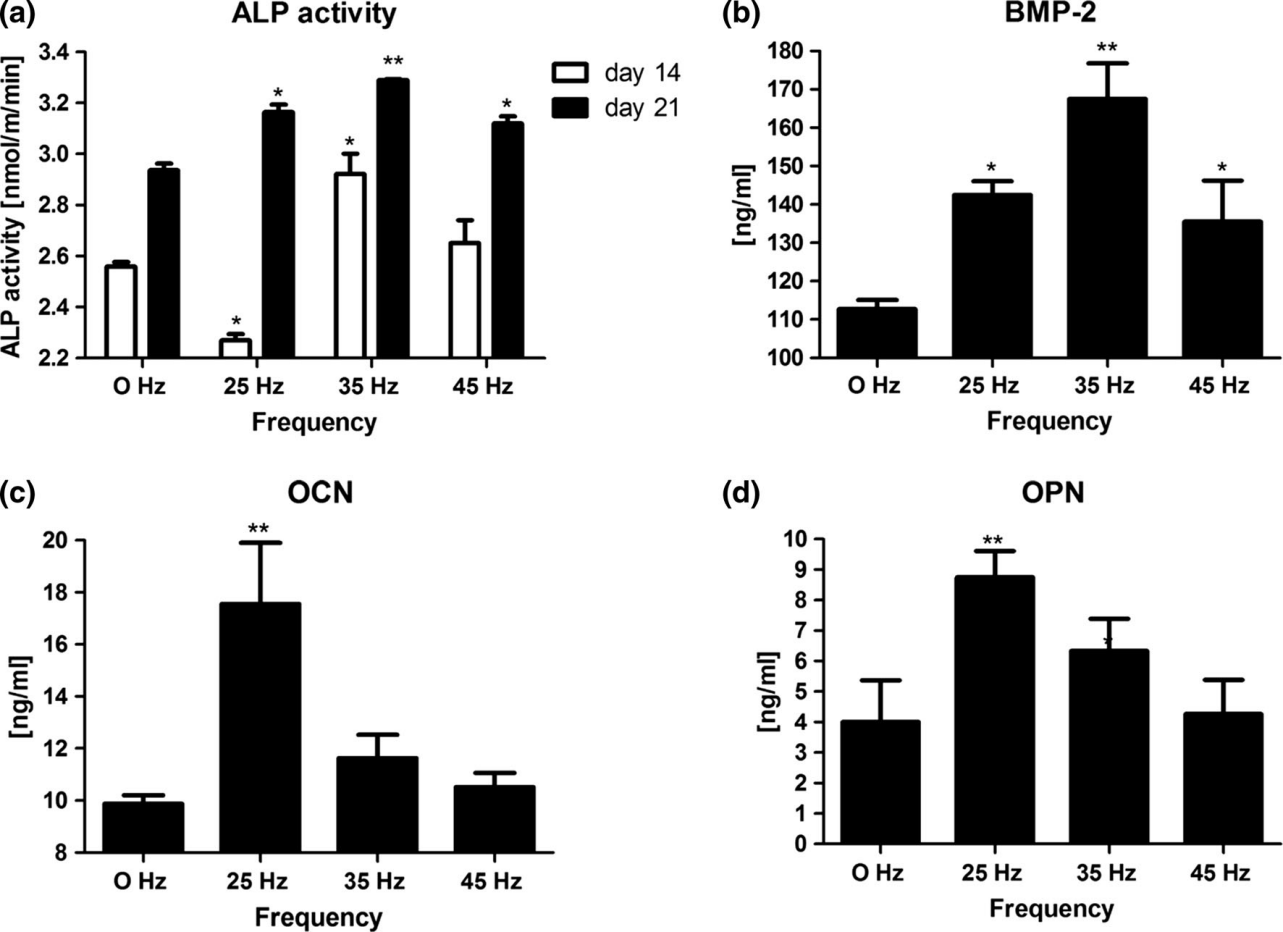
Alkaline phosphatase activity (a) and ELISA: bone morphogenetic protein 2 (b), osteocalcin (c) and osteopontin (d). *p < 0.05, **p < 0.01, all groups vs. control culture.

Osteogenic differentiation was higher in all groups exposed to vibrations as compared to controls without vibration application. In cells exposed to 25 Hz vibration, ALP activity at 14 days was lower (2.26 ± 0.2) and at 21 days higher (3.19 ± 0.1) than controls (Fig. 7a). In cells exposed to 35 Hz vibration, ALP activity was higher at both 14 and 21 days (2.26 ± 0.4; 3.3 ± 0.1, respectively) as compared to control (Fig. 7a). In cells exposed to 45 Hz vibration, ALP activity at 14 and 21 days was higher (2.68 ± 0.1; 3.09 ± 0.1, respectively) as compared to control (Fig. 7a). Concentration of BMP-2 at protein level measured after 21 days of culture was higher in all investigated groups as compared to control. The highest level of BMP-2 was observed in group propagated to 35 Hz vibrations (Fig. 7b). However, the highest levels of OCN and OPN were observed in cells exposed to 25 Hz vibration in comparison to other groups. (Figs. 7c and 7d).

**Figure 8:**
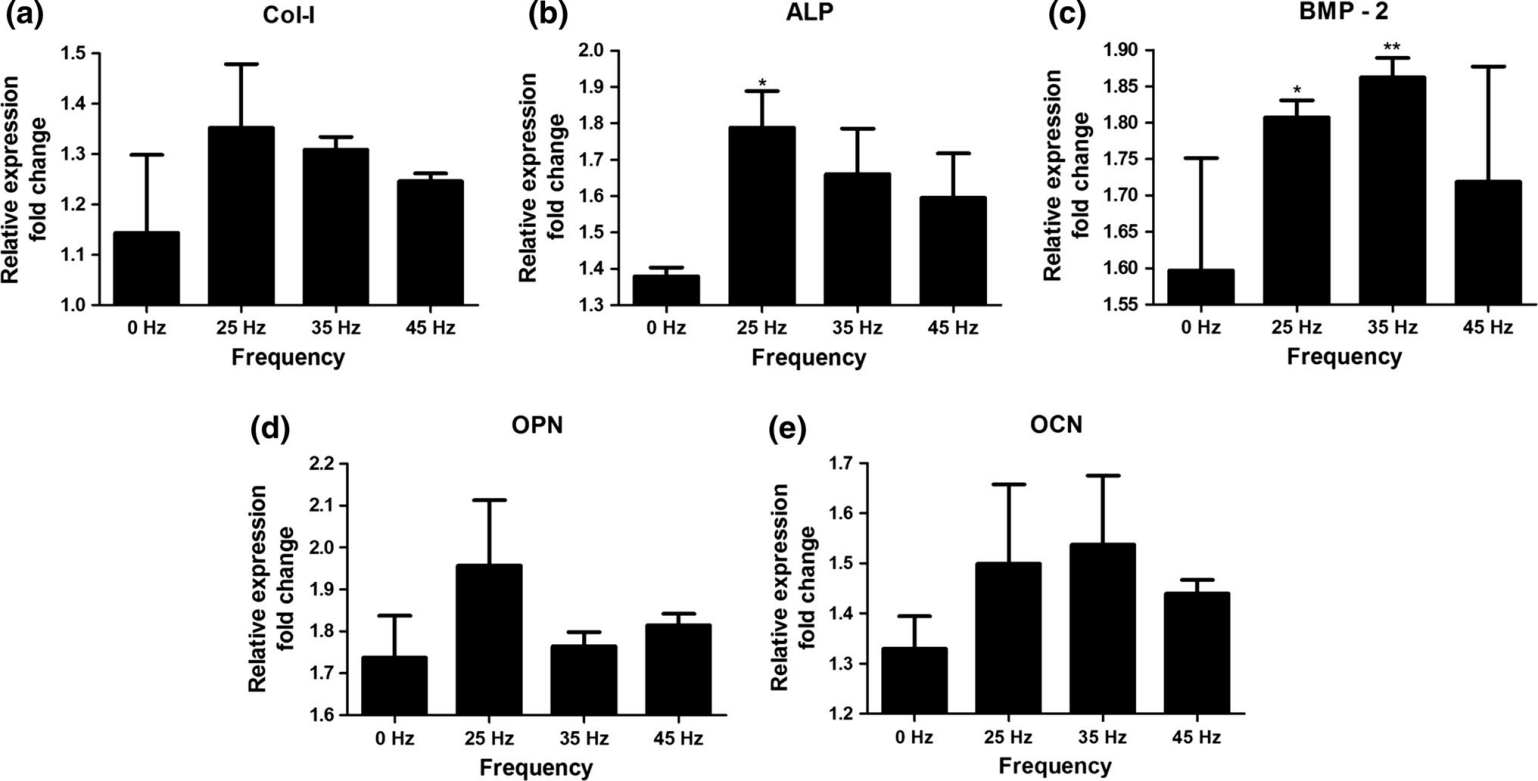
Gene expression: collagen type I, alkaline phosphatase, bone morphogenetic protein 2, osteopontin, osteocalcin. *p < 0.05, **p < 0.01, all groups vs. control culture.

Cells exposed to 25 Hz vibration had the highest level of gene expression of Collagen type I, ALP and OPN, whereas cells exposed to 35 Hz vibration had the highest expression levels of BMP-2 and OCN (Fig. 8).

### Analysis of Integrin Expression in Response to Vibration Stimulation

**Figure 9:**
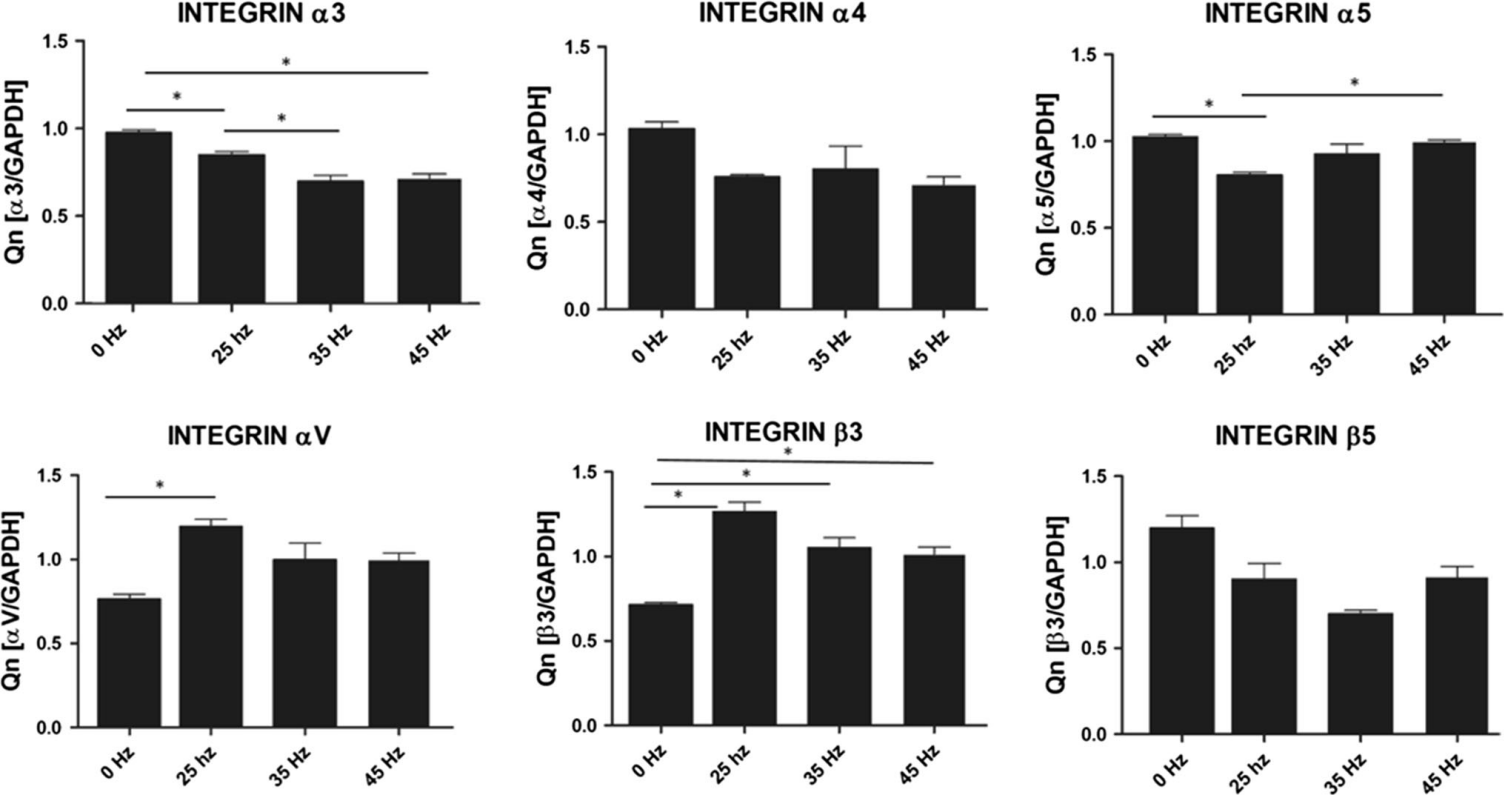
Integrin expression: α3, α4, α5, αV, β3, β5. *p < 0.05, all groups vs. control culture.

To sense and translate the applied external mechanical signals, cells express mechanoreceptors on their surface, such as integrins. In our study qPCR analysis demonstrated a slight increase of integrin αV and β3 subunit expression after 35 Hz stimulation in comparison to control (0 Hz) (Fig. 9). We also found that when cells were stimulated with 25 Hz vibrations, hASCs significantly upregulated integrin αV and β3 subunit. Interestingly, after 25 Hz stimulation, the highest increase in expression of the β3 integrin was observed. With respect to integrin subunits α3, α4, α5 and β5 expression levels were similar between to stimulated groups, however down-regulated as compared to control.

## Discussion

Mechanical vibration has been shown to promote bone tissue regeneration by enhancing bone formation, increasing bone strength, and decreasing bone loss.^10^,^11^ The underlying mechanisms responsible for this effect are not yet fully understood. Mechanical stimulation, in the form of vibrations, is thought to induce changes by causing distortions in the cell’s cytoskeleton. Sudden deformation of the cells leads to accelerated fluid flow and activates a cascade of secondary messengers.^3^,^37^

The aim of the present study was to investigate whether mechanical stimulation with LMLF vibration enhances osteogenic differentiation potential of hASCs obtained from elderly subjects. During the 21-days of stimulation, we observed increased osteogenic differentiation, especially in hASCs exposed to 25 Hz vibration. These findings suggest that this form of mechanical stimulation could be used as an adjunct in cell-based therapies to improve their effectiveness when used to treat problematic large bone defects.

In the current study, we observed that the frequency of the vibration had an important influence on cell morphology, proliferation, and osteogenic differentiation, all functions essential in the bone healing process. Based on this observation, when considering the use of vibrations as an adjunct in the treatment of bone defects, it is important that the vibration frequency be calibrated. The device we built for this study fits these criteria. The designed device is simple to operate and generates continuous, pulsed, or combined signal, low frequency vibrations.

In the current research, we observed that none of the three vibration frequencies to which we exposed the cells caused a decrease in hASC proliferative activity. Of all the frequencies tested, 25 and 35 Hz caused the greatest increase in proliferative activity as compared to controls. The hASCs exposed to 25 Hz vibration demonstrated a significantly higher PF and consequently the lowest PDT, compared to the other groups. The cells in this group generated nearly threefold greater amounts of calcium, sixfold greater amounts of phosphorus, and generated hydroxyapatite-like structures that were approximately 2.5-fold greater in diameter than in the control group. In addition, cells exposed to 25 Hz vibrations secreted the greatest amount of OCN and OPN, which was confirmed by gene expression analysis. This group was also characterized by the highest expression of Col-I, confirming advanced levels of osteogenesis.

Interestingly, cells exposed to 35 Hz vibrations secreted more BMP-2 and showed higher ALP activity than the group stimulated with 25 Hz vibrations. The down regulated ALP activity on day 14 in cells exposed to 25 Hz vibrations might have been due to the fact that the expression of ALP increases with osteoblast maturation, and then decreases with mineralization of osteoid, as was demonstrated in previous studies.^17^

Cells exposed to 45 Hz vibrations showed slightly lower proliferative activity, and less osteogenic differentiation than cells exposed to lower frequencies, though still greater that the non-stimulated controls.

In the current study, we decided to use an osteogenic medium as a model for vibration stimulations, to maximize conditions that are recognized for *in vivo* tests. Bearing in mind these facts, the synergistic effect of the vibration cannot be totally excluded. However, our results still deliver valuable information concerning the potential of vibrations of frequencies *per se* during the osteogenic differentiation process.

Further research, exposing other types of cells to the same frequencies to see if they can be stimulated to differentiate into other cell lineages would be interesting. In previous studies, Pre *et al*.^34^^–^ 36 using a comparable frequency (30 Hz) but a tenfold higher magnitude (3 g), and longer vibration duration (45 min), reported similar results with sarcoma osteogenetic cells (SAOS-2), human adipose derived stem cells (hASC), and human bone marrow derived stem cells (hBMSC). In their studies, as in the present study, they observed that Osteocalcin, Collagen type I, and ALP expression was highest in cells exposed to 30 Hz. We saw the same in our 25 and 35 Hz groups.

While Pre *et al*. described a decrease in proliferation activity in their stimulated cells, other researchers reported no changes in cell number or viability^40^ or, as in the present study, described a significantly higher PF.^12^ Interestingly, in a study by Kim *et al*.^12^ which used a similar method to the one we used in this study, they observed that bone marrow derived hMSC differentiated into osteoblasts after vibration stimulation. These findings demonstrate that both bone marrow-derived and adipose-derived MSCs exposed to similar vibration frequencies show similar differentiation activity. This observation may present an opportunity to replace difficult to obtain bone marrow derived-MSCs with more easily obtained hASCs.

Analysis of up-regulation of integrin family might be explained the positive effect of LFLM on the osteogenic differentiation. In our research, we observed statistical significance up-regulation of integrin αV and β3 subunits on the mRNA level after 25 Hz stimulation. Furthermore, we observed up-regulation of integrin β3 subunit in 35 and 45 Hz stimulated cells that also was statistical significance. Interestingly, in a study by Martino *et al*.^21^ they observed that integrin α5 subunit has a crucial role in control of MSC osteogenic differentiation. Here, we found that in all frequencies, influence down-regulation of integrin α5 subunit. Moreover, we observed that LFLM down regulates integrin α3, α4 and β5 expression. Our research might be a new view on the effect LFLM on osteogenic differentiation.

Our findings suggest that vibration frequency may be an important factor in controlling cell differentiation. Depending on the frequency, cells can be induced to differentiate into different cell lineages. We showed that 25 Hz was the optimal frequency for inducing the formation of cell structures typical for osteogenesis—e.g., osteogenic nodules, hydroxyapatite, and the highest level of specific proteins and genes. While it remains problematic to change the expression of genes to increase regeneration process of tissues, low frequency mechanical and electrical signals are relatively easy to control and implement and protect tissues from unexpected reactions. Bone adaptation is driven mostly by rather dynamic^40^ however higher frequencies could stimulate cells to differentiation into adipose tissue and altered the balance between fat and bone.

## Conclusions

Our results indicate that LMLF (25 Hz) vibrations stimulate proliferation and osteogenic differentiation of hASCs. These findings could potentially be used to optimize tissue engineering cell-based treatments of non-healing bone defects, especially in elderly populations.
